# Supplementary figures and images for: Vacuole pH loss triggers ESCRT-dependent plasma membrane remodeling to prevent amino acid toxicity
Source: bioRxiv. 2026 Jun 6:2026.06.05.730386. Preprint. [Version 1] doi: 10.64898/2026.06.05.730386 (PMC13252423; doi:10.64898/2026.06.05.730386)

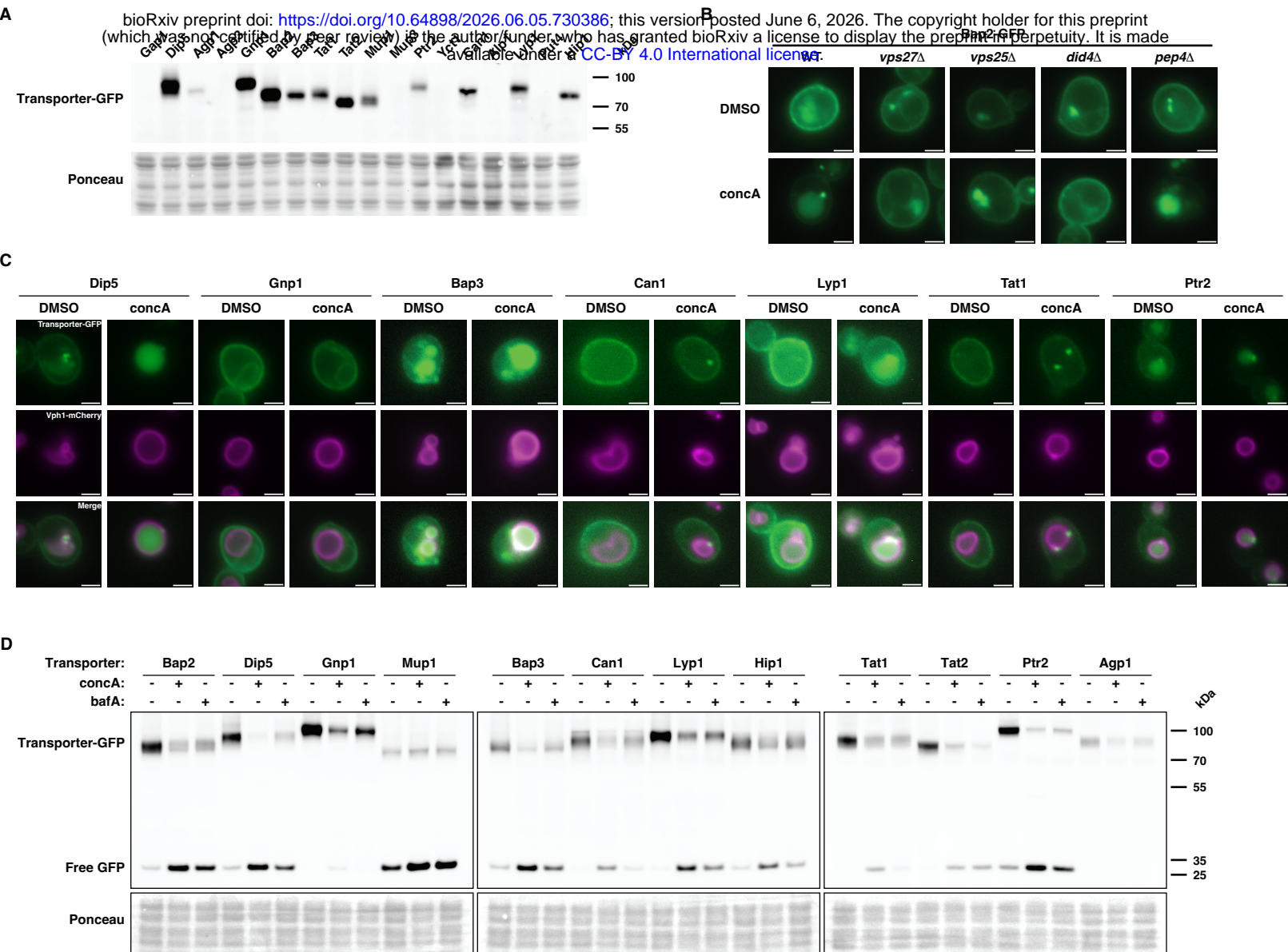

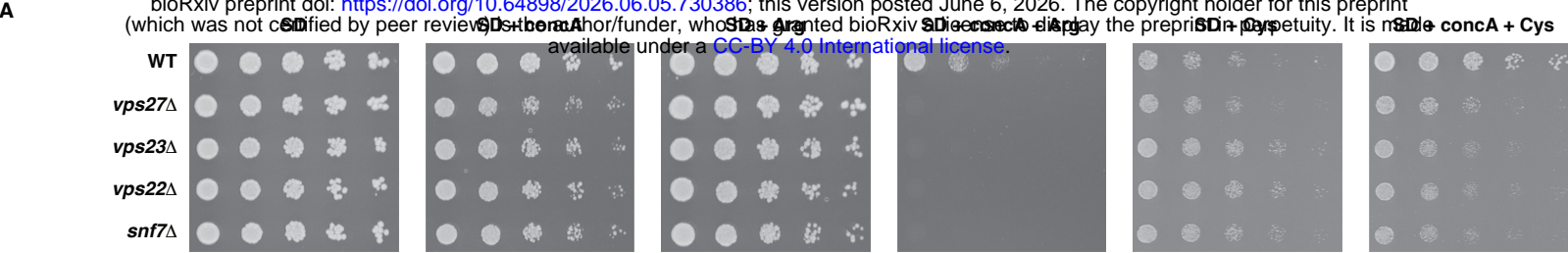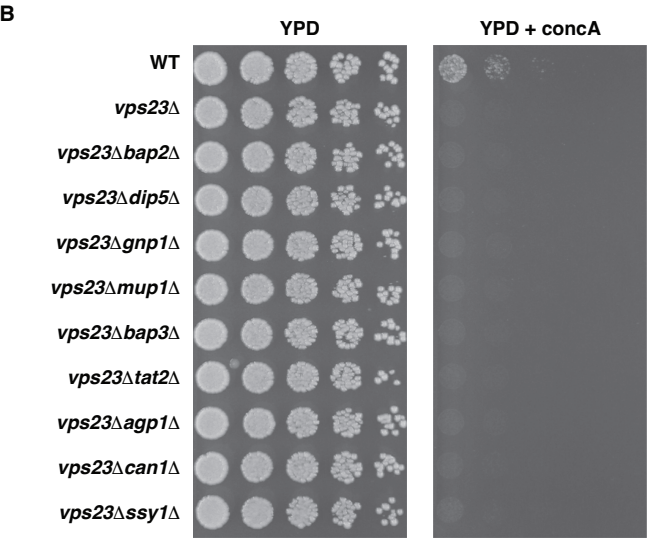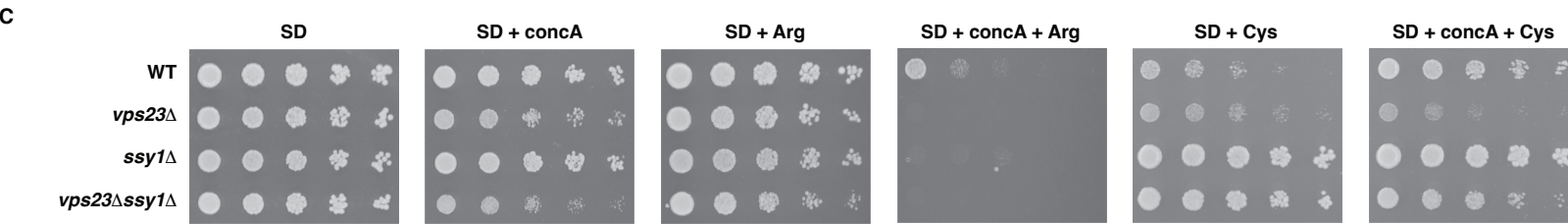

Supplemental Figure 2

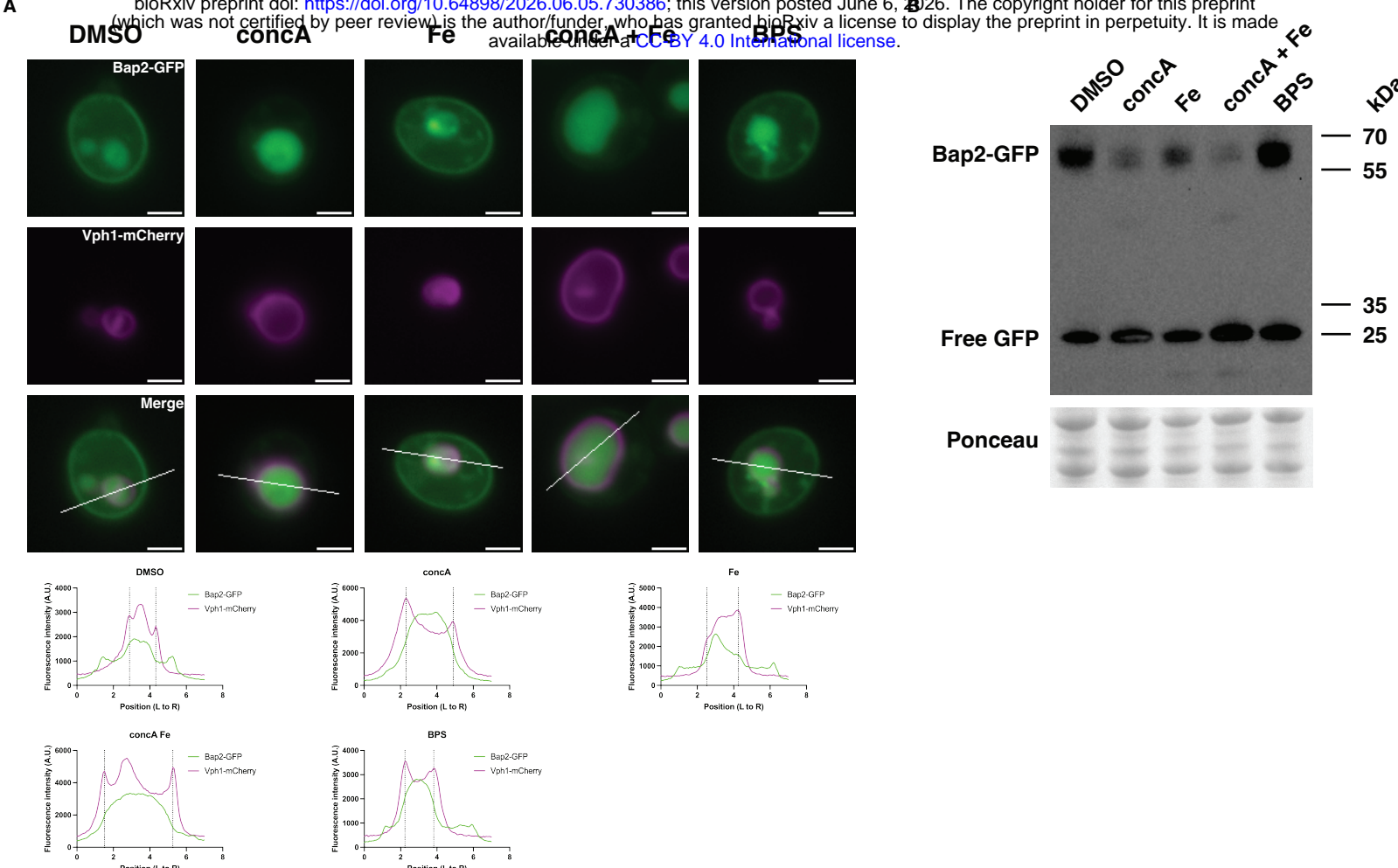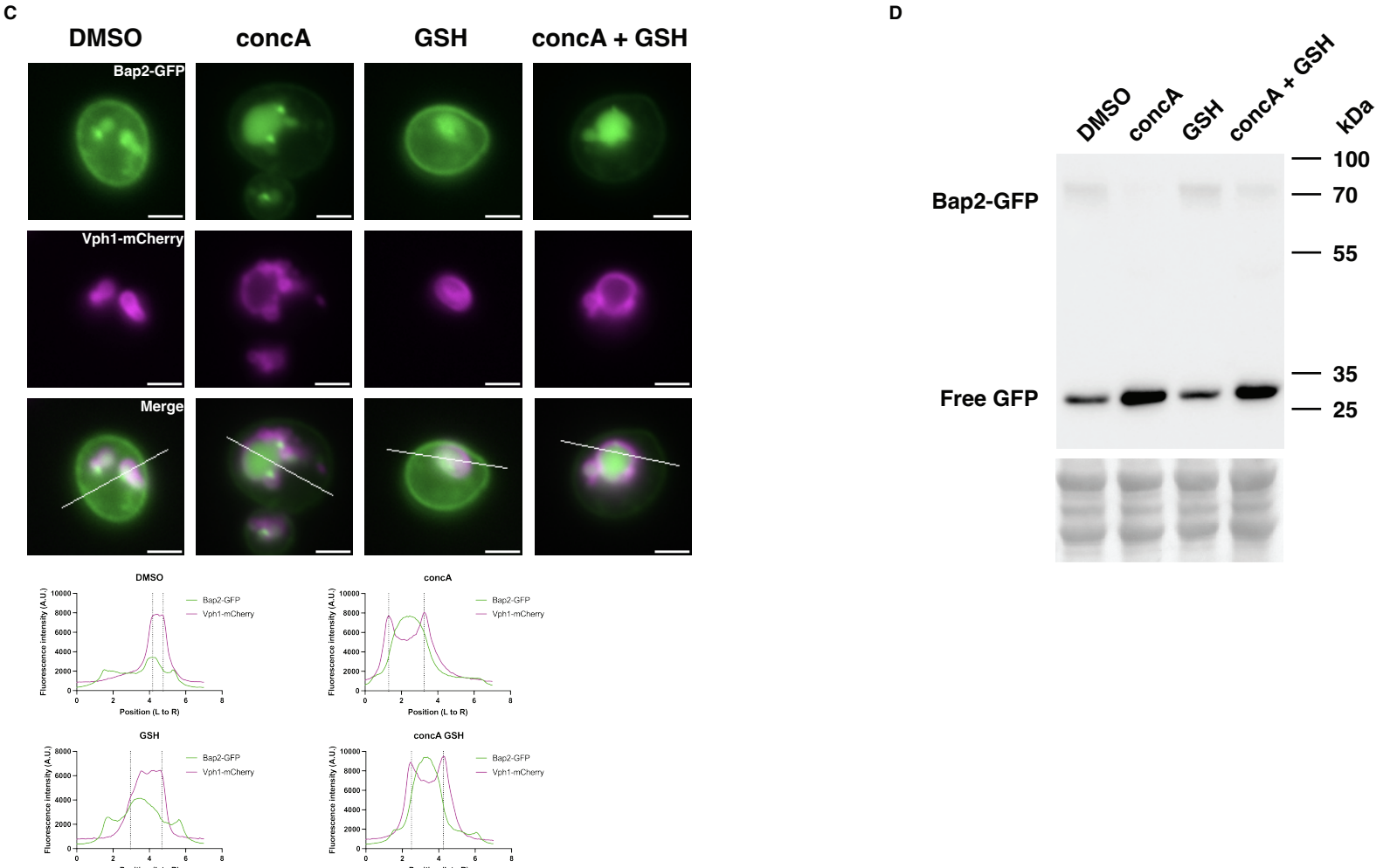

Supplemental Figure 3

Supplement: Supplement 5 — Supplemental Figure 1. Vacuole deacidification triggers broad rerouting of plasma membrane amino acid transporters to the vacuole (related to Figure 2) (A) Western blot analysis of exponentially growing strains expressing GFP-tagged plasma membrane amino acid transporters (AATs) in YPD medium. Cells were harvested from exponentially growing cultures. Primary antibody: α-GFP. (B) Microscopy of Bap2–GFP localization following 3 h treatment with DMSO or concA in WT, ESCRT mutant, or pep4Δ strains. Scale bars: 2 μm. (C) Microscopy showing localization of additional GFP-tagged plasma membrane amino acid transporters following 3 h treatment with DMSO or concA. Scale bars: 2 μm. (D) Western blot analysis of GFP-tagged plasma membrane amino acid transporters following 3 h treatment with DMSO, concA, or bafilomycin A1 (bafA). Cells were harvested from exponentially growing cultures. Primary antibody: α-GFP. Supplemental Figure 2. Amino acid availability drives synthetic lethality between vacuole deacidification and ESCRT disruption (related to Figure 3) (A) Spot dilution growth assays of WT and ESCRT mutant strains grown on SD medium containing DMSO or concA and supplemented with arginine (7 mM) or cysteine (7 mM). (B) Spot dilution growth assays of WT, vps23Δ, single plasma membrane amino acid transporter deletion strains in the vps23Δ background, and vps23Δ ssy1Δ strains grown on YPD containing DMSO or concA. (C) Spot dilution growth assays of WT, vps23Δ, ssy1Δ, and vps23Δ ssy1Δ strains grown on SD medium containing DMSO or concA and supplemented with arginine (7 mM) or cysteine (7 mM). Supplemental Figure 3. Iron limitation and oxidative stress do not drive transporter rerouting during vacuole stress (related to Figure 4) (A) Microscopy of Bap2–GFP following 3 h treatment with DMSO, concA, iron supplementation, or the iron chelator BPS in YPD medium. Scale bars: 2 μm. (B) Western blot analysis corresponding to (A). Primary antibody: α-GFP. (C) Microscopy of Bap2–GFP follo [file NIHPP2026.06.05.730386v1-supplement-5.pdf]
